# Whole-Genome Sequence of the Ammonia-Oxidizing Bacterium Nitrosomonas stercoris Type Strain KYUHI-S, Isolated from Composted Cattle Manure

**DOI:** 10.1128/MRA.00742-19

**Published:** 2019-08-22

**Authors:** Tatsunori Nakagawa, Yuki Tsuchiya, Reiji Takahashi

**Affiliations:** a Department of Chemistry and Life Science, College of Bioresource Sciences, Nihon University, Fujisawa, Kanagawa, Japan; University of Southern California

## Abstract

This work reports the complete genome sequence of a chemoautotrophic ammonia-oxidizing bacterium, Nitrosomonas stercoris strain KYUHI-S^T^ (= ATCC BAA-2718^T^ and NBRC 110753^T^). The assembled genome is composed of a circular chromosome and a large plasmid.

## ANNOUNCEMENT

*Nitrosomonas* is a genus of Gram-negative ammonia-oxidizing bacteria (AOB) widely distributed in soil, wastewater treatment plants, freshwater environments, and marine environments (1). The AOB Nitrosomonas stercoris type strain KYUHI-S was isolated from composted cattle manure (2) and is an aerobic betaproteobacterium belonging to the *Nitrosomonas* cluster 7 (1). The environmental DNAs related to *N. stercoris* are frequently found in strongly eutrophic environments, such as wastewater treatment plants, animal manure, and municipal solid waste landfills (2).

*N. stercoris* type strain KYUHI-S was incubated for 6 days at 30°C in 4 liters of modified 2-[4-(hydroxyethyl)-1-piperazinyl] ethanesulfonic acid-trace element medium (2). The nucleic acid was extracted using NucleoBond buffer set III and an AXG 500 column (Macherey-Nagel) following the manufacturer’s instructions. The genome of *N. stercoris* was sequenced using the MiSeq (Illumina) and GridION (Oxford Nanopore Technologies) platforms. A Nextera DNA flex library prep kit (Illumina) was used for a MiSeq library, and MiSeq paired-end reads were generated using a MiSeq reagent kit v3 (600 cycles; Illumina). A GridION library was prepared using the native barcoding expansion (EXP-NBD104) and a ligation sequencing kit and was sequenced with an R9.4.1 flow cell (all Oxford Nanopore Technologies). The quality of the Illumina reads was assessed with Sickle v1.33 (3) using a minimum quality value (QV) score of <20 and a minimum nucleotide length of <127, resulting in 746,120 reads (coverage, 173.37×). An adapter from the Nanopore reads was removed using Porechop v0.2.3 (4) (default parameters), resulting in 849,935 raw reads covering 11,759,933,494 sequenced bases with an *N*_50_ value of 30,109 bp. The quality of the Nanopore reads was assessed with Filtlong v0.2.0 (https://github.com/rrwick/Filtlong) using a minimum nucleotide length of <1,000 to yield 235,000,000 bp and with Canu v1.8 (5) using the “-correct” and “-nanopore-raw” parameters, resulting in 3,764 reads (coverage, 5,066.93×). The filtered Illumina and Nanopore reads were assembled with Unicycler v0.4.7 (6) (default parameters) and resulted in a total genome size of 2,382,370 bp with a G+C content of 44.8%. Both the *N*_50_ contig size and the longest contig size were 2,320,919 bp. An assembly graph was visualized with Bandage v0.8.1 (7) (default parameters), showing a circular chromosome (2,320,919 bp) and a circular plasmid (61,451 bp) similar to the plasmid pNeutP1 of Nitrosomonas eutropha C91 (8). Genome completeness and contamination were estimated with CheckM v1.0.12 (9) (default parameters), showing that the completeness was 99.03% and the contamination was 0.12%. Subsequently, the assembled genome sequence was annotated with Prokka v1.13 (10) using the “-rfam” and “-genus, -species, -strain” parameters, which resulted in 2,274 coding sequences (CDSs) with a single copy of the 16S-23S-5S rRNA operon and 40 tRNA genes in the chromosome and 99 CDSs in the plasmid.

Genes involved in ammonia oxidization to nitrite, nitrous oxide production from nitrite, and carbon fixation were found in the chromosome. Genes encoding multidrug resistance proteins and lipopolysaccharide export proteins (11) were also found in the chromosome.

### Data availability.

The complete genome sequence of *N. stercoris* strain KYUHI-S^T^ has been deposited in DDBJ under the accession numbers AP019755 and AP019756, and in the DDBJ Sequence Read Archive (DRA) under accession number DRA008477.
